# The dataset about the deformations under axial compression of concrete and cement-sand mortar impregnated with oil

**DOI:** 10.1016/j.dib.2018.10.152

**Published:** 2018-11-02

**Authors:** Alexander P. Svintsov, Svetlana L. Shambina

**Affiliations:** Peoples׳ Friendship University of Russia (RUDN University), 6 Miklukho-Maklaya Street, Moscow 117198, Russian Federation

**Keywords:** Concrete, Cement-sand mortar, Industrial oil I-30A, Corn oil, Olive oil

## Abstract

This article contains data of longitudinal and transverse deformations of concrete and cement-sand prisms impregnated with mineral I-30A, corn and olive oil, under axial compression up to loads that do not create destructive stresses. The stress level, as the ratio of the compressive stress *σ* to the design compressive strength *R*_*b*_, varied from 0.054 *σ/R*_*b*_ to 0.845 *σ/R*_*b*_. The values of deformation for various axial compression loads are presented for control samples and for samples impregnated with mineral oil I-30A with a viscosity of 15 °E, corn oil with a viscosity of 9 °E, and olive oil with a viscosity of 11 °E on Engler scale. The data are associated with the research article “Influence of viscosity of vegetable and mineral oil on deformation properties of concrete and cement-sand mortar” (Alexander et al., 2018).

**Specifications table**

**Table:** 

Subject area	*Civil engineering*
More specific subject area	*The construction of buildings*
Type of data	*Tables*
How data was acquired	*Axial compression of the samples was carried out on a 600 kN hydraulic testing machine at a speed of 7 kN/min. Measurement of deformations was carried out with indicators having additional accessories with an accuracy of 0.0001 mm.*
Data format	*The data for the control samples correspond to the three measurements for each stage of axial compression. The data for the samples impregnated with oil, correspond to the five measurements for each stage of axial compression. The magnitude of axial compression did not reach the value causing the destruction of the samples. The data in the tables do not have statistical processing.*
Experimental factors	*Concrete samples are impregnated with oil with different viscosity after the strength set. The deformations of the samples under axial compression are experimentally determined*
Experimental features	*Deformations are measured under axial compression. The stresses from axial compression did not exceed the strength of concrete*
Data source location	*Peoples׳ Friendship University of Russia* (*RUDN University*)*. 6 Miklukho-Maklaya Street, Moscow, 117198, Russian Federation*
Data accessibility	*Data is within this article*
Related research article	[1]*Alexander P. Svintsov, Svetlana L. Shambina, Influence of viscosity of vegetable and mineral oil on deformation properties of concrete and cement-sand mortar, Construction and Building Materials. 190* (*2018*) *964-974.*

**Value of the data**

• The data can be used to develop models for assessing the reliability and technical safety of concrete supporting structures of industrial buildings.
• The data can be used for comparison with data on the effect of other types of oil on concrete and cement-sand elements of building structures.
• This data can be used to solve engineering problems of increasing the reliability of concrete building structures.
• Data can be used in the course of structures’ selection for industrial buildings as part of the design.
• These data can be used with other sets of similar data to develop general models of the effect of oil on concrete and cement-sand mortar.

## 1 Data

This data article presents the values of longitudinal and transverse deformations under axial compression of concrete and cement-sand samples impregnated with mineral and vegetable oils. 36 prisms were made of concrete and cement-sand mortar. Three prisms made of concrete and three prisms made of cement-sand mortar were used as control samples. Five prisms made of concrete and five prisms made of cement-sand mortar were used for each type of oil: mineral oil I-30A, corn oil and olive oil. These oils have the following viscosity according to the Engler scale: for mineral oil I-30A it is 15 °E, for corn oil it is 9 °E, for olive oil it is 11 °E. The tables contain values of deformation at each stress level from 0.054 *σ/R*_*b*_ to 0.845 *σ/R*_*b*_. The stress level is the ratio of the axial compression stress *σ* to the design compressive strength *R*_*b*_. The values of concrete samples׳ deformations under various axial compression loads are presented in Table 1, Table 2, Table 3, Table 4, Table 5, Table 6. The values of deformations for the samples made of the cement-sand mortar at the different loads of axial compression are presented in Table 7, Table 8, Table 9, Table 10, Table 11, Table 12. This dataset is associated with a research article entitled “Influence of viscosity of vegetable and mineral oil on deformation properties of concrete and cement-sand mortar” (Svintsov et al., 2018) [1].

**Table 1:** Transverse deformations, *ε*_*tr*_ × 10^−^^4^ (mm), of concrete samples impregnated with olive oil at various axial compression loads *σ/R*_*b*_.

(*σ/R*_*b*_)	Сontrol samples			Impregnated with olive oil				
0.00	0.00	0.00	0.00	0.00	0.00	0.00	0.00	0.00
0.054	0.10	0.10	0.10	0.20	0.10	0.20	0.18	0.18
0.110	0.11	0.10	0.08	0.44	0.45	0.45	0.52	0.46
0.170	0.20	0.25	0.12	0.53	0.58	0.72	0.89	0.68
0.270	0.24	0.21	0.22	0.78	0.81	0.72	0.96	0.82
0.330	0.37	0.36	0.42	0.97	1.70	0.77	1.15	1.15
0.390	0.53	0.49	0.56	1.16	1.74	1.10	1.62	1.41
0.510	0.79	0.75	0.85	1.75	2.20	1.54	1.70	1.79
0.570	1.16	1.12	1.15	1.60	2.42	2.15	2.10	2.07
0.680	1.44	1.50	1.35	2.25	2.90	1.90	3.10	2.53
0.730	1.91	2.50	1.22	3.50	3.70	3.50	2.30	3.25
0.790	2.37	2.70	2.50	3.45	3.12	4.12	4.70	3.85
0.840	3.10	3.60	2.40	4.90	6.20	3.95	4.90	4.98
0.845	4.20	4.20	4.00	6.70	7.60	5.11	6.90	6.57

**Table 2:** Transverse deformations, *ε*_*tr*_ × 10^−4^ (mm), of concrete samples impregnated with corn oil at various axial compression loads *σ/R*_*b*_.

(*σ/R*_*b*_)	Сontrol samples			Impregnated with corn oil				
0.00	0.00	0.00	0.00	0.00	0.00	0.00	0.00	0.00
0.054	0.10	0.10	0.10	0.11	0.08	0.17	0.20	0.14
0.110	0.11	0.10	0.08	0.40	0.36	0.22	0.44	0.35
0.170	0.20	0.25	0.12	0.42	0.48	0.62	0.59	0.52
0.270	0.24	0.21	0.22	0.78	0.62	0.72	0.96	0.77
0.330	0.37	0.36	0.42	0.82	1.20	0.54	1.15	0.92
0.390	0.53	0.49	0.56	0.95	1.12	1.60	0.76	1.11
0.510	0.79	0.75	0.85	1.54	1.90	1.12	1.10	1.42
0.570	1.16	1.12	1.15	1.45	1.90	1.80	1.70	1.72
0.680	1.44	1.50	1.35	1.75	2.10	2.20	2.40	2.11
0.730	1.91	2.50	1.22	2.80	2.40	3.60	2.40	2.80
0.790	2.37	2.70	2.50	3.00	3.12	3.82	4.00	3.48
0.840	3.10	3.60	2.40	3.80	5.20	4.60	4.40	4.50
0.845	4.20	4.20	4.00	5.10	6.50	5.50	6.90	6.00

**Table 3:** Transverse deformations, *ε*_*tr*_ × 10^−^^4^ (mm), of concrete samples impregnated with mineral oil I-30A at various axial compression loads *σ/R*_*b*_.

(*σ/R*_*b*_)	Сontrol samples			Impregnated with industrial oil I-30A				
0.00	0.00	0.00	0.00	0.00	0.00	0.00	0.00	0.00
0.054	0.10	0.10	0.10	0.10	0.08	0.17	0.06	0.10
0.110	0.11	0.10	0.08	0.12	0.19	0.11	0.07	0.12
0.170	0.20	0.25	0.12	0.20	0.23	0.11	0.27	0.20
0.270	0.24	0.21	0.22	0.55	0.52	0.72	0.42	0.55
0.330	0.37	0.36	0.42	0.84	0.90	0.50	1.15	0.85
0.390	0.53	0.49	0.56	1.18	1.12	1.60	0.76	1.16
0.510	0.79	0.75	0.85	1.50	2.20	1.25	1.10	1.51
0.570	1.16	1.12	1.15	1.80	1.90	1.80	1.70	1.80
0.680	1.44	1.50	1.35	2.20	2.10	2.20	2.40	2.22
0.730	1.91	2.50	1.22	2.90	2.40	3.80	2.40	2.87
0.790	2.37	2.70	2.50	3.58	2.90	3.82	4.00	3.57
0.840	3.10	3.60	2.40	4.80	5.40	4.60	4.40	4.80
0.845	4.20	4.20	4.00	6.70	7.00	5.50	7.60	6.70

**Table 4:** Longitudinal deformations, *ε*_*lo*_ × 10^−^^4^ (mm), of concrete samples impregnated with olive oil at various axial compression loads *σ/R*_*b*_.

(*σ/R*_*b*_)	Сontrol samples			Impregnated with olive oil				
0.00	0.00	0.00	0.00	0.00	0.00	0.00	0.00	0.00
0.054	0.70	0.30	1.10	0.10	0.12	0.15	0.07	0.09
0.110	0.97	0.80	1.15	0.13	0.17	0.10	0.12	0.13
0.170	1.60	2.10	1.10	0.62	0.54	0.51	0.69	0.75
0.270	2.34	2.98	1.70	0.96	0.75	1.15	1.05	0.92
0.330	3.35	2.10	4.60	1.74	1.62	1.94	2.22	1.21
0.390	4.25	5.40	3.10	2.51	2.15	2.62	3.73	1.55
0.510	5.15	4.10	6.20	3.69	3.30	3.64	4.20	3.65
0.570	6.30	7.40	5.20	4.05	3.80	5.30	3.94	3.18
0.680	7.40	8.60	6.20	5.35	5.20	6.10	5.40	4.72
0.730	8.55	9.90	7.20	6.72	6.50	7.20	7.00	6.20
0.790	10.20	12.50	7.90	8.15	7.52	7.80	8.80	8.30
0.840	11.80	13.40	10.20	8.97	9.40	8.20	9.10	9.20
0.845	13.55	15.20	11.90	10.37	10.20	8.20	10.90	12.20

**Table 5:** Longitudinal deformations, *ε*_*lo*_ × 10^−4^ (mm), of concrete samples impregnated with corn oil at various axial compression loads *σ/R*_*b*_.

(*σ/R*_*b*_)	Сontrol samples			Impregnated with corn oil				
0.00	0.00	0.00	0.00	0.00	0.00	0.00	0.00	0.00
0.054	0.70	0.30	1.10	0.10	0.05	0.06	0.12	0.17
0.110	0.97	0.80	1.15	0.10	0.09	0.17	0.06	0.12
0.170	1.60	2.10	1.10	0.60	0.99	0.22	0.59	0.97
0.270	2.30	2.90	1.70	1.00	0.85	1.03	1.21	0.92
0.330	3.30	2.10	4.60	1.30	1.26	1.58	1.15	1.44
0.390	4.20	5.40	3.10	1.70	2.15	1.90	1.60	1.40
0.510	5.10	4.10	6.20	3.10	2.84	3.44	2.75	3.70
0.570	6.30	7.40	5.20	4.10	4.14	3.51	3.90	5.24
0.680	7.40	8.60	6.20	4.50	3.40	4.40	5.15	5.22
0.730	8.50	9.90	7.20	5.90	5.50	6.55	5.20	6.40
0.790	10.20	12.50	7.90	7.40	7.60	6.10	8.20	7.70
0.840	11.80	13.40	10.20	8.82	8.90	8.40	9.10	8.90
0.845	13.55	15.20	11.90	9.725	9.20	8.50	11.20	10.00

**Table 6:** Longitudinal deformations, *ε*_*lo*_ × 10^−4^ (mm), of concrete samples impregnated with mineral oil I-30A at various axial compression loads *σ/R*_*b*_.

(*σ/R*_*b*_)	Сontrol samples			Impregnated with industrial oil I-30A				
0.00	0.00	0.00	0.00	0.00	0.00	0.00	0.00	0.00
0.054	0.70	0.30	1.10	0.10	0.15	0.07	0.05	0.13
0.110	0.97	0.80	1.15	0.39	0.25	0.44	0.36	0.54
0.170	1.60	2.10	1.10	0.73	0.55	1.10	0.90	0.40
0.270	2.34	2.98	1.70	1.30	1.80	1.20	1.20	1.00
0.330	3.35	2.10	4.60	1.60	1.20	2.00	1.70	1.60
0.390	4.25	5.40	3.10	2.50	3.20	2.00	2.40	2.40
0.510	5.15	4.10	6.20	3.35	3.10	3.10	3.80	3.40
0.570	6.30	7.40	5.20	4.25	4.70	4.50	4.00	3.80
0.680	7.40	8.60	6.20	5.15	4.80	5.10	5.40	5.30
0.730	8.55	9.90	7.20	5.72	5.90	6.10	5.10	5.80
0.790	10.20	12.50	7.90	7.43	8.20	7.60	7.55	6.40
0.840	11.80	13.40	10.20	8.60	8.20	9.40	9.20	7.70
0.845	13.55	15.20	11.90	10.10	10.20	8.10	12.10	10.30

**Table 7:** Transverse deformations, *ε*_*tr*_ × 10^−4^ (mm), of cement-sand mortar samples impregnated with olive oil at various axial compression loads *σ/R*_*b*_.

(*σ/R*_*b*_)	Сontrol samples			Impregnated with olive oil				
0.00	0.00	0.00	0.00	0.00	0.00	0.00	0.00	0.00
0.054	0.14	0.11	0.16	0.37	0.36	0.40	0.33	0.37
0.11	0.27	0.30	0.24	0.74	0.70	0.80	0.75	0.72
0.17	0.42	0.40	0.44	1.20	1.30	1.40	1.00	1.10
0.27	1.00	0.80	1.20	2.30	2.50	2.00	2.10	2.60
0.33	1.18	1.25	1.10	2.68	3.20	2.50	2.10	2.90
0.39	1.75	1.60	1.90	3.13	3.30	3.00	3.40	2.80
0.51	2.25	2.30	2.20	3.73	4.20	3.50	3.40	3.80
0.57	2.30	2.60	2.00	4.75	4.00	5.20	5.00	4.80
0.68	3.55	3.60	3.50	5.73	5.50	6.70	5.40	5.30
0.73	3.80	4.00	3.60	6.65	6.90	6.20	6.50	7.00
0.79	4.35	4.40	4.30	7.65	8.00	7.20	7.60	7.80
0.84	5.45	5.90	5.00	10.08	11.20	9.60	10.00	9.50
0.845	6.90	7.60	6.20	11.28	11.60	11.20	11.00	11.30

**Table 8:** Transverse deformations, *ε*_*tr*_ × 10^−4^ (mm), of cement-sand mortar samples impregnated with corn oil at various axial compression loads *σ/R*_*b*_.

(*σ/R*_*b*_)	Сontrol samples			Impregnated with corn oil				
0.00	0.00	0.00	0.00	0.00	0.00	0.00	0.00	0.00
0.054	0.14	0.11	0.16	0.33	0.30	0.36	0.35	0.32
0.11	0.27	0.30	0.24	0.52	0.48	0.55	0.56	0.49
0.17	0.42	0.40	0.44	0.74	0.79	0.70	0.77	0.70
0.27	1.00	0.80	1.20	1.62	1.40	1.70	1.60	1.80
0.33	1.18	1.25	1.10	1.80	1.90	1.60	1.80	1.90
0.39	1.75	1.60	1.90	2.40	2.50	2.30	2.60	2.20
0.51	2.25	2.30	2.20	2.95	3.20	2.60	2.80	3.20
0.57	2.30	2.60	2.00	3.75	3.80	4.10	3.70	3.40
0.68	3.55	3.60	3.50	4.85	5.40	4.90	4.20	4.90
0.73	3.80	4.00	3.60	5.50	6.00	6.20	4.80	5.00
0.79	4.35	4.40	4.30	6.17	6.00	6.30	5.40	7.00
0.84	5.45	5.90	5.00	7.65	8.20	7.50	7.20	7.70
0.845	6.90	7.60	6.20	8.40	8.20	8.10	9.60	7.70

**Table 9:** Transverse deformations, *ε*_*tr*_ × 10^−4^ (mm), of cement-sand mortar samples impregnated with mineral oil I-30A at various axial compression loads *σ/R*_*b*_.

(*σ/R*_*b*_)	Сontrol samples			Impregnated with industrial oil I-30A				
0.00	0.00	0.00	0.00	0.00	0.00	0.00	0.00	0.00
0.054	0.14	0.11	0.16	0.36	0.31	0.39	0.33	0.42
0.11	0.27	0.30	0.24	0.66	0.70	0.62	0.63	0.70
0.17	0.42	0.40	0.44	0.84	0.80	0.83	0.83	0.88
0.27	1.00	0.80	1.20	1.80	2.10	1.60	2.00	1.50
0.33	1.18	1.25	1.10	2.10	2.50	2.20	2.00	1.70
0.39	1.75	1.60	1.90	2.58	2.70	2.40	2.40	2.80
0.51	2.25	2.30	2.20	3.30	3.30	3.60	3.40	2.90
0.57	2.30	2.60	2.00	4.15	4.00	4.60	4.20	3.80
0.68	3.55	3.60	3.50	5.13	5.00	4.90	5.60	5.00
0.73	3.80	4.00	3.60	5.93	6.20	5.60	6.00	5.90
0.79	4.35	4.40	4.30	6.73	6.70	5.90	7.00	7.30
0.84	5.45	5.90	5.00	8.13	8.10	7.60	8.80	8.00
0.845	6.90	7.60	6.20	9.13	9.10	9.60	8.80	9.00

**Table 10:** Longitudinal deformations, *ε*_*lo*_ × 10^−4^ (mm), of cement-sand mortar samples impregnated with olive oil at various axial compression loads *σ/R*_*b*_.

(*σ/R*_*b*_)	Сontrol samples			Impregnated with olive oil				
0.00	0.00	0.00	0.00	0.00	0.00	0.00	0.00	0.00
0.054	0.92	0.83	1.00	0.12	0.10	0.09	0.15	0.14
0.11	1.42	1.34	1.50	0.12	0.09	0.14	0.16	0.10
0.17	2.18	2.25	2.10	0.56	0.44	0.62	0.57	0.60
0.27	3.16	3.33	3.00	0.92	0.91	0.95	0.97	0.84
0.33	4.25	3.80	4.70	1.20	1.15	1.35	1.11	1.20
0.39	6.03	6.25	5.80	1.98	1.50	2.50	2.20	1.70
0.51	7.15	7.30	7.00	3.03	3.10	3.70	2.80	2.50
0.57	8.58	8.40	8.75	4.28	4.30	4.80	4.10	3.90
0.68	10.33	10.20	10.45	5.35	5.36	5.80	4.90	5.36
0.73	11.95	12.15	11.75	6.95	6.20	7.20	7.40	7.00
0.79	13.85	13.80	13.90	8.11	8.25	8.00	7.90	8.30
0.84	15.39	14.90	15.87	8.58	8.60	9.00	8.20	8.50
0.845	19.38	18.75	20.00	9.12	9.25	8.80	9.30	9.12

**Table 11:** Longitudinal deformations, *ε*_*lo*_ × 10^−^^4^ (mm), of cement-sand mortar samples impregnated with corn oil at various axial compression loads *σ/R*_*b*_.

(*σ/R*_*b*_)	Сontrol samples			Impregnated with corn oil				
0.00	0.00	0.00	0.00	0.00	0.00	0.00	0.00	0.00
0.054	0.92	0.83	1.00	0.11	0.11	0.15	0.09	0.10
0.11	1.42	1.34	1.50	0.13	0.13	0.10	0.15	0.09
0.17	2.18	2.25	2.10	0.77	0.88	0.67	0.75	0.85
0.27	3.16	3.33	3.00	1.75	1.60	1.90	2.00	1.50
0.33	4.25	3.80	4.70	2.42	2.20	2.60	2.00	2.90
0.39	6.03	6.25	5.80	3.28	3.30	3.90	2.70	3.50
0.51	7.15	7.30	7.00	4.16	4.40	4.00	3.90	4.90
0.57	8.58	8.40	8.75	5.72	5.20	6.80	5.50	5.30
0.68	10.33	10.20	10.45	6.70	6.40	7.20	6.50	6.70
0.73	11.95	12.15	11.75	8.58	8.80	9.20	7.90	8.70
0.79	13.85	13.80	13.90	10.10	11.30	9.10	10.00	9.20
0.84	15.39	14.90	15.87	11.20	11.50	10.70	11.00	11.40
0.845	19.38	18.75	20.00	13.38	13.50	13.00	14.00	13.30

**Table 12:** Longitudinal deformations, *ε*_*lo*_ × 10^−4^ (mm), of cement-sand mortar samples impregnated with mineral oil I-30A at various axial compression loads *σ/R*_*b*_.

(*σ/R*_*b*_)	Сontrol samples			Impregnated with industrial oil I-30A				
0.00	0.00	0.00	0.00	0.00	0.00	0.00	0.00	0.00
0.054	0.92	0.83	1.00	0.09	0.10	0.09	0.10	0.09
0.11	1.42	1.34	1.50	0.16	0.15	0.20	0.10	0.12
0.17	2.18	2.25	2.10	0.55	0.54	0.55	0.60	0.40
0.27	3.16	3.33	3.00	1.54	1.30	1.60	1.70	1.50
0.33	4.25	3.80	4.70	1.92	2.10	1.80	2.20	1.60
0.39	6.03	6.25	5.80	2.76	2.50	3.50	2.40	2.60
0.51	7.15	7.30	7.00	4.25	4.00	6.00	3.75	3.75
0.57	8.58	8.40	8.75	4.92	4.92	4.92	4.92	4.92
0.68	10.33	10.20	10.45	6.20	6.20	6.20	6.20	6.20
0.73	11.95	12.15	11.75	7.75	7.75	7.75	7.75	7.75
0.79	13.85	13.80	13.90	8.64	8.64	8.64	8.64	8.64
0.84	15.39	14.90	15.87	9.85	9.85	9.85	9.85	9.85
0.845	19.38	18.75	20.00	10.40	10.40	10.40	10.40	10.40

## 2 Experimental design, materials and methods

The data were obtained by axial compression of samples made of concrete and cement-sand mortar in course of an experimental study. The standard values of resistance to axial compression (prism strength) and to axial tension, a description of the experiment, machines and measuring devices, materials and their proportions are presented in Ref. [1].
