# Supplementary material for: The dataset about the deformations under axial compression of concrete and cement-sand mortar impregnated with oil
Source: Data Brief. 2018 Nov 2;21:1363–9. doi: 10.1016/j.dib.2018.10.152 (PMC6234250; doi:10.1016/j.dib.2018.10.152)
Supplement: Supplementary file 1 — Supplementary material [file mmc1.docx]

**Conflict of interest form**

Conflict of interest form: none.
